# Using physiologically based models to predict *in vivo* skeletal muscle energetics

**DOI:** 10.1242/jeb.249966

**Published:** 2025-03-31

**Authors:** Ryan N. Konno, Glen A. Lichtwark, Taylor J. M. Dick

**Affiliations:** ^1^ School of Biomedical Sciences, The University of Queensland, St Lucia, QLD 4072, Australia; ^2^ School of Exercise and Nutrition Sciences, Queensland University of Technology, Brisbane, QLD 4000, Australia

**Keywords:** Biomechanics, Metabolic cost, Mathematical modelling, Muscle–tendon mechanics

## Abstract

Understanding how muscles use energy is essential for elucidating the role of skeletal muscle in animal locomotion. Yet, experimental measures of *in vivo* muscle energetics are challenging to obtain, so physiologically based muscle models are often used to estimate energy use. These predictions of individual muscle energy expenditure are not often compared with indirect whole-body measures of energetic cost. Here, we examined and illustrated the capability of physiologically based muscle models to predict *in vivo* measures of energy use, which rely on fundamental relationships between muscle mechanical state and energy consumption. To improve model predictions and ensure a physiological basis for model parameters, we refined our model to include data from isolated muscle experiments and account for inefficiencies in ATP recovery processes. Simulations were performed to capture three different experimental protocols, which involved varying contraction frequency, duty cycle and muscle fascicle length. Our results demonstrated the ability of the model to capture the dependence of energetic cost on mechanical state across contractile conditions, but tended to underpredict the magnitude of energetic cost. Our analysis revealed that the model was most sensitive to the force–velocity parameters and the data informing the energetic parameters when predicting *in vivo* energetic rates. This work highlights that it is the mechanics of skeletal muscle contraction that govern muscle energy use, although the precise physiological parameters for human muscle likely require detailed investigation.

## Teaser

**Summary:** Experimental measures of muscle energy consumption are difficult to obtain during *in vivo* conditions; however, physiologically based mathematical models can predict muscle energy use *in vivo* based on muscle mechanical state.

## INTRODUCTION

Knowledge of how muscles consume energy to develop forces and perform mechanical work is fundamental in understanding the mechanisms that govern neuromuscular function and locomotor performance. Predicting energy consumption requires knowledge of not only the physiological processes that govern muscle energetics at the level of the fibre (Barclay and Curtin, 2023), but also the structure of the muscle and connective tissue (Curtin and Barclay, 2023), the interactions with the external environment (Collins et al., 2015; Lichtwark and Wilson, 2007) and the neural control strategies used to perform a movement (Lai et al., 2018; Falisse et al., 2019). An improved capacity to predict energetics during the diverse movements that humans and other animals perform, and whether energy is optimized under certain conditions, would afford insights into the mechanisms behind muscle energetic cost in a variety of locomotor tasks.

The energy used by skeletal muscle to perform mechanical work comes from chemical potential energy stored in ATP molecules; however, inefficiencies occur in the hydrolysis and rephosphorylization of ATP that result in the production of heat (Woledge et al., 1985). To obtain measures of the heat released from muscle during contractions, experiments are typically performed on isolated animal muscle, whereby the heat can be directly measured using a thermopile (e.g. Woledge et al., 1985; Barclay, 1996). Although thermopile experiments have provided us with invaluable information on the mechanisms governing muscle energetics at the muscle fibre level, these data are not easily extrapolated to *in vivo* human muscle. The reason we cannot extrapolate to the contractile conditions observed *in vivo* is that they often occur at submaximal activation levels and are dynamic in nature, with time-varying muscle fibre lengths and shortening rates; conditions different to the controlled contractile environment of isolated muscle experiments. Our understanding of *in vivo* muscle energetics can be advanced using mathematical models in combination with experimental data (Umberger and Rubenson, 2011). Modelling approaches are advantageous as they allow us to investigate direct cause and effect mechanisms, which is not often possible through experimental techniques.

A variety of approaches have been used to model the energetics of skeletal muscle during contraction. At the microscopic, single sarcomere level, Huxley-based models (Huxley, 1957) capture the dynamics of individual actin–myosin cross-bridges. While providing a detailed biophysical model that can be scaled up to a whole-muscle level (van Soest et al., 2019), there are limited experimental data to inform the models, which may limit further insights beyond more phenomenological models. At the whole-body level, models have been developed based on measures of energetics from indirect calorimetry using O_2_ and CO_2_ measurements (e.g. Kram and Taylor, 1990; Cavagna et al., 1977). Although these models are able to sufficiently capture changes in net whole-body energetic cost, their highly phenomenological nature limits their potential to investigate the physiological principles that underlie muscle energetics and they are not transferable to predict movements beyond the original experimental protocol. Alternatively, physiologically based, but still phenomenological models (e.g. Lichtwark and Wilson, 2005; Bhargava et al., 2004; Umberger et al., 2003) serve as a middle ground, utilizing rich datasets from single muscle experiments where fibre lengths and heat rates are directly measured. Within these models, the energy rates owing to maintenance, activation and shortening/lengthening heats, as well as work, are included (for more detail, see Barclay and Curtin, 2023). The physiologically based models have been applied to whole-body musculoskeletal simulations of movement, such as walking (Umberger, 2010; Miller, 2014; Koelewijn et al., 2019) and hopping (Farris et al., 2014), but there can be large differences between model predictions and experimental measures of energy use (Miller, 2014; Koelewijn et al., 2019); for example, during walking, the predicted cost of transport varied from 2.45 J m^−1^ kg^−1^ in the Bhargava et al. (2004) model to 7.15 J m^−1^ kg^−1^ in the Lichtwark and Wilson (2007) model, whereas the measured cost was 3.35 J m^−1^ kg^−1^ (Miller, 2014). The differences in energetic cost could be due to inaccuracies of either the rate constants governing the energetic rate or the muscle dynamics that primarily determine the energetic rate. The muscle dynamics, including individual muscle forces, activation and length changes, are difficult to accurately capture during dynamic tasks such as walking. Even if the energetics model is sufficiently accurate, errors propagated by the mechanical model could result in erroneous energetic costs. During typical locomotor tasks, it is particularly challenging to accurately predict the high shortening rates and varying muscle activation levels, which are known to alter muscle energetics (Wakeling et al., 2023), as well as handle the agonist and antagonist co-contraction that results in incorrect muscle states (Uchida and Delp, 2021). Thus, it could be more informative to investigate mechanisms governing energy use in simplified fixed-angle single joint tasks, where the muscle state can be measured.

The purpose of this study was to investigate the role that changes to muscle mechanical state have on the energetic cost of muscle contraction and demonstrate the ability of the model to capture these changes. A physiologically based model of muscle contraction was used that predicts energetic cost based on the muscle state, which allows for isolation of direct cause–effect relationships linking mechanics and energetics. Further, these models are built on mechanistic principles at the muscle level and do not require fitting of a conceptual model based on muscle outputs, for example, the rate of force generation (Doke and Kuo, 2007), which often require optimization to experimental data (see e.g. van der Zee and Kuo, 2021). The fitting of the data required by these models limits their applicability to a specific movement. To avoid tuning parameters to fit the energetic costs when comparing with experimental *in vivo* contractions, we utilized physiological data obtained via direct measurements of energy use in isolated fibre-bundles from Barclay (1996). Although similar models of energy consumption during contraction have been investigated previously for concentric and eccentric contractions (Umberger et al., 2003; Lentz-Nielsen et al., 2023), this study focuses on isometric muscle–tendon unit conditions to identify the mechanisms governing muscle energy consumption. To do this, we adapted and compared the model from Lichtwark and Wilson (2005, 2007) with a series of human experiments that investigated single joint contractions *in vivo* (van der Zee and Kuo, 2021; Beck et al., 2020, 2022), where the whole-body energy use was measured using indirect calorimetry. By capturing the energetics from *in vivo* experiments, we aimed to identify whether the assumed factors (activation level and strain rates) contributing to energetic cost in a modified energetics model based on Lichtwark and Wilson (2005, 2007) could explain changes to energetic cost with changes to the contractile condition: rate of force development (van der Zee and Kuo, 2021), duty cycle (Beck et al., 2020) and initial muscle fascicle length (Beck et al., 2022). We extend the model from Lichtwark and Wilson (2005, 2007) by accounting for fibre type muscle properties, as well as the metabolic recovery processes. Further, as model parameters are not known exactly, we performed a sensitivity analysis on the model parameters to understand how the quality of experimental data impacts model predictions.

## MATERIALS AND METHODS

**Fig. 1.**
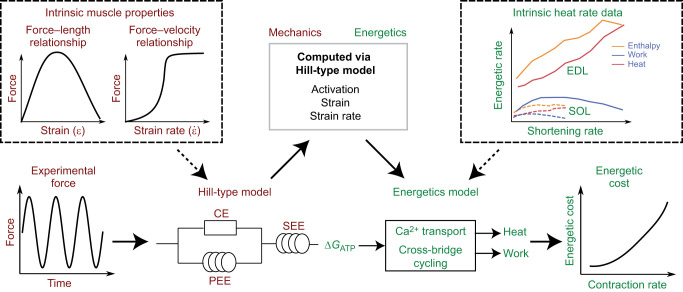
**Overview of the modelling approach.** For the experiments investigated in this study (van der Zee and Kuo, 2021; Beck et al., 2020, 2022), we used functions based on the experimentally measured forces as input to the Hill-type model. We then utilized a one-dimensional Hill-type model to solve for activation, strain and strain rate. These variables were used as inputs to an energetics model, which is based on Lichtwark and Wilson (2005, 2007). The heat rates are based on experimental data from Barclay (1996) in single muscle preparations of slow soleus (SOL) and fast extensor digitorum longus (EDL) muscles. The energetic cost of muscle contraction was then computed to determine the effect of the given experimental conditions: changes to contraction frequency (van der Zee and Kuo, 2021), duty cycle (Beck et al., 2020) or muscle fascicle length (Beck et al., 2022). CE, contractile element; SEE, series elastic element; PEE, parallel elastic element; Δ*G*_ATP_, free energy of ATP.

Our mathematical model consists of a mechanical Hill-type model in combination with the energetics model based on Lichtwark and Wilson (2005, 2007). The mechanical component of the model was used to predict the muscle mechanics based on the forces measured within the experiment, and then the energetics component used the mechanical data to predict the whole muscle energetic cost (Fig. 1). The model parameters were refined to capture physiological data from Barclay (1996). The mechanical and energetics models were implemented in Python and are available at https://github.com/ryankonno/KLD2024-EnergeticsModel.

### Mechanical model

We utilized a Hill-type muscle model that consists of a contractile element (CE) along with an in series elastic element (SEE), such that the total force from the muscle–tendon unit is given by
(1)

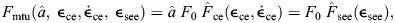
where 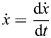, with 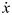 representing a given variable. Here, *F*_mtu_ is the total force from the muscle–tendon unit, 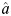 is the normalized activation in the muscle and *F*_0_ is the maximum isometric force. The normalized force from the contractile element, 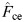, and series elastic element, 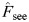, are written as a function of strains (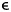
_ce_ and 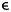
_see_) and the strain rate 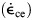. We neglected the presence of a parallel elastic element commonly seen in Hill-type models, as the experiments were performed at isometric whole muscle–tendon unit lengths, whereby the muscle did not lengthen past optimal length (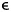
_ce_≤0). In these cases, there would be no contributions from the classic parallel elastic element (as implemented in Zajac, 1989) or minimal contributions from parallel forces that arise due to differences between slack length and optimal muscle length (Lieber et al., 2025). The function 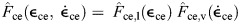 defines the intrinsic force–length and force–velocity properties of the muscle model, and uses the formulation from Dick et al. (2017).

### Energetics model

We formulate the total energy rate of the muscle in terms of the muscle heat rate and work rate (Lichtwark and Wilson, 2005, 2007). We used this model as our basis, but with the modification of the maintenance and shortening heat rate constants according to data from Barclay (1996), along with the addition of a recovery heat term. These changes were made to obtain an energetics model that is entirely based on physiologically measured parameters. Here, the rates are normalized by *F*_0_*l*_0_, where *l*_0_ is the resting fascicle length, to give normalized units of s^−1^. We first compute the total energy rate, 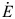, required for the contraction as a combination of initial (the energy required for pumping of Ca^2+^, cross-bridge cycling and heat released during the cycling of phosphocreatine), 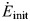, and recovery heat rates (the energy lost during the generation of ATP), 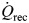:
(2)

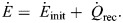


The initial energy rate from the muscle is given as
(3)

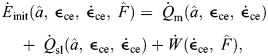
where 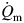 is the maintenance heat rate, 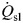 is the shortening and lengthening heat rate, and 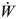 is the work rate. Here, we use the term maintenance heat for what is typically referred to as the combination of heat from activation and generation of isometric force from the cross-bridges (see Barclay and Curtin, 2023 for a review). 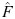 is the muscle force normalized to maximum isometric force, *F*_0_. The maintenance heat rate is given by
(4)

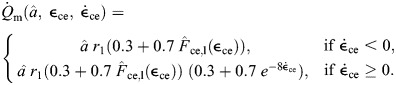
*r*_1_ is a parameter determined by experimental data from Barclay (1996) with units of s^−1^. 
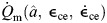
 represents the heat associated with Ca^2+^ flow and cross-bridge cycling under isometric conditions, but is scaled to account for decreased heat rates in lengthening muscle (Linari et al., 2003). In addition to the isometric heat rates, there are typically increased heat rates associated with shortening and lengthening muscle (Hill, 1938; Barclay, 1996; Linari et al., 2003), which are given by
(5)

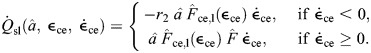
*r*_2_ is the unitless shortening heat rate parameter. In lengthening muscle there is no additional heat from cross-bridge cycling as is seen in shortening (Barclay and Curtin, 2023); however, while the muscle lengthens, there is negative work being done on the muscle. Not all of this work is transferred into elastic potential energy as the process is not completely efficient. Our model assumes that all work done on the muscle is eventually released as heat, as has been shown in experiments (Linari et al., 2003; Barclay and Curtin, 2023). In addition to the initial heat rates, there is also the rate of work done by the contractile element, which is given by
(6)

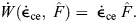


In addition to the initial heat and work rates, this model includes contributions from the recovery processes. Here, we intend to model the steady-state metabolic process, which should largely be due to aerobic pathways and occur at a constant efficiency. We assume that 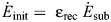, where ε_rec_ is the efficiency of the metabolic pathway and 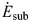 is the energy conversion rate of a given substrate (e.g. glucose). Thus, the ratio between the recovery heat rate and the initial enthalpy can be represented by a constant, *r*_rec_, giving us
(7)

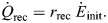


A summary of the variables and parameters used in the model, along with their sources, is given in Table 1. All of the constants used in the model are based on experimental physiological data and are not optimized to the experimental tasks described below.

**Table 1.** Summary of model variables and parameters with definitions and units

Variable	Definition	Source
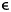 _ce_	Strain of the CE	Calculated based on experiments (see Results)
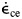	Strain rate of the CE (s^−1^)	Calculated from ε_ce_
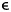 _see_	Strain of the SEE	Solved for in the Hill-type model
*t*	Time (s)	Simulation length is based on experiment (see Results)
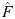	Total muscle force (normalized by *F*_0_)	Calculated using Eqn 1
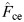	CE force (normalized by *F*_0_)	Calculated using properties from Dick et al. (2017)
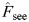	SEE force (normalized by *F*_0_)	Calculated depending on the experiment (see Results)
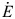	Total energy rate of the muscle (s^−1^)	Calculated using Eqn 2
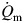	Maintenance heat rate (s^−1^)	Calculated using Eqn 4
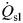	Shortening/lengthening heat rate (s^−1^)	Calculated using Eqn 5
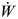	Work rate (s^−1^)	Calculated using Eqn 6
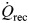	Recovery heat rate (s^−1^)	Calculated using Eqn 7
Parameter	Definition	Source
*l*_0_	Resting length of muscle (m)	Varied depending on experiment (see Results)
*F*_0_	Maximum force at *l*_0_ (N)	Calculated based on PCSA from Handsfield et al. (2014)
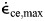	Maximum strain rate (s^−1^)	Estimated from experimental values from Dick et al. (2017)
κ	Force–velocity curvature	Slow-fibre values from Dick et al. (2017)
*k*	Tendon stiffness (N m^−1^)	Calculated depending on the experiment (see Results)
*r*_1_	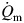 parameter (s^−1^)	Optimised from Barclay (1996) (see Eqn 4)
*r*_2_	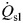 parameter (s^−1^)	Optimised from Barclay (1996) (see Eqn 5)
ε_rec_	Efficiency of ATP generation	Values from Barclay (2019)
*r*_rec_	Ratio of initial enthalpy to recovery heat	Values from Barclay (2019)
*s*_f_	Shift in muscle fibre length (m)	Defined based on data from Beck et al. (2022)

CE, contractile element; SEE, series elastic element. Parameters used in this study were based on physiological data and not optimised to the experimental condition.

### Model parameters

The behaviour of the above model depends on *r*_1_ and *r*_2_, which are the constants in the maintenance and shortening heat rates, respectively. To determine the energetic parameters, we used existing data on the thermodynamic behaviour of muscle during shortening contractions. Barclay (1996) measured both muscle work and heat rates in single muscles consisting mainly of either slow- or fast-type fibres. The slow fibre-type data are from experiments on mouse soleus (SOL), whereas the fast fibre data are from mouse extensor digitorum longus (EDL). To determine our model parameters, we minimized the relative error between predicted model heat rates and the experimental data (see Supplementary Materials and Methods for more detail). We first scaled the experimental data to account for temperature differences. The experiment was conducted at 25°C, whereas *in vivo* temperatures are near 35°C. Thus to account for temperature, the heat rate parameters were increased by a factor of 4 (Rall and Woledge, 1990) and the maximum strain rate 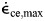 by a factor of 2 (Ranatunga, 1998). The resulting parameters are shown in Table 2. The recovery heat rates are dependent on the efficiency of the metabolic processes, and we assume *r*_rec_=1 (Barclay, 2019) for all the simulations used within this study. Here, we avoid fitting model parameters to the experimental condition, because, although improving energetic predictions, it will make the model less generalizable and not provide meaningful insights relating model mechanics to energetic cost.

**Table 2.** Values for the energetic parameters, *r*_1_ (s^−1^) and *r*_2_ (unitless)

	*r*_1_	*r*_2_
Slow-type muscle (SOL)	0.618	0.234
Fast-type muscle (EDL)	2.792	0.697

The parameters were obtained via fit to physiological data from Barclay (1996). *r*_1_ and *r*_2_ are the maintenance heat and shortening heat parameters, respectively.

### Computational experiments

To test the model, we compared predictions of muscle energy consumption with net whole-body energy consumption obtained via indirect calorimetry. We set up our model to capture three different experimental paradigms that investigated the influence of (i) rate of force development (van der Zee and Kuo, 2021), (ii) duty cycle (Beck et al., 2020) and (iii) muscle fascicle length (Beck et al., 2022) on muscle energetics. The first study, van der Zee and Kuo (2021), demonstrated that the energetic cost increased with faster cycle frequencies during a seated knee extension task. Beck et al. (2020) examined the effect of duty cycle, the fraction of the contraction cycle where the muscle is actively producing force, on energetics during plantarflexion contractions with knee flexed to 50 deg to isolate the contribution from the SOL. They found that energetic cost increased as duty cycle decreased. Beck et al. (2022) explored the effect of fascicle length, owing to differences in ankle angle during cyclical isometric plantarflexion contractions, on energetic cost. They found that an increased ankle angle corresponded to shorter fascicle lengths, which led to increases in energy consumption. These experimental studies were chosen to evaluate our model predictions because they elicit different energetic responses and each measured relevant parameters that influence muscle energy use, namely muscle activation level (via surface EMG) and fascicle length changes of at least one of the muscles contracting (via B-mode ultrasound imaging). We will refer to the three simulations as vdZ2021, B2020 and B2022 for the respective experimental conditions. The specific model parameter values used for each of these experiments are provided in the Supplementary Materials and Methods.

### van der Zee and Kuo (2021) simulation

**Fig. 2.**
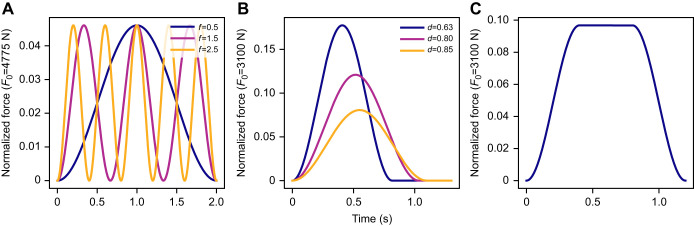
**Experimentally based force used as input to the Hill-type model.** (A) van der Zee and Kuo (2021) (vdZ2021) simulation input force trace, which varies with the frequency of contraction (*f*). (B) Beck et al. (2020) (B2020) simulation input force trace used to investigate the effect of duty cycle (*d*), while maintaining the same integral of the force with respect to time by increasing the peak force. (C) Beck et al. (2022) (B2022) simulation input force trace, which maintains the same force input across conditions while decreasing muscle fascicle lengths.

van der Zee and Kuo (2021) investigated the effect of contraction frequency on the energetic cost of isometric muscle contraction. In their experiment, participants were seated and performed simultaneous bilateral knee isometric extensions following a sinusoidal torque trace, at frequencies ranging from 0.5 to 2.5 Hz (Fig. 2A). The main driver behind changes in net whole-body energetic cost is assumed to be the knee extensor muscles. Thus, we implemented our model as a lumped muscle model for the combined vastus lateralis, vastus intermedius, vastus medialis and rectus femoris; for one lumped muscle, we would expect it to match half of the total energetic cost reported. We used the *l*_0_ value from the vastus lateralis of *l*_0_=0*.*095 m (van der Zee and Kuo, 2021) for the lumped muscle, because strains and activation levels are reported in van der Zee and Kuo (2021) for this muscle, and we used *F*_0_=4775 N based on physiological cross-sectional area (PCSA) calculated in Handsfield et al. (2014). The tendon stiffness of the model (*k*=1*.*58×10^4^ N m^−1^) was prescribed to achieve experimentally measured fascicle strains over a cycle (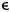
_max_=−0*.*147). As we assumed a linear tendon stiffness, it is possible to solve explicitly for tendon stiffness at maximum muscle strain. Starting with a sinusoidal torque matching the experimental conditions, we solved the Hill-type model for the time-varying strains, strain rates and activation levels (Fig. 1). The outputs from the Hill-type model were then used by the energetics model to predict the energetic cost. Given the low activation levels observed for these contractions, we assumed the energetic and force–velocity parameters for slow-type fibres during these simulations, as the quadriceps have approximately 50% slow and 50% fast fibres (Feiereisen et al., 1997).

### Beck et al. (2020) simulation

The Beck et al. (2020) experiments investigated the influence of duty cycle on energetic cost. Here, we used the duty cycles reported in the experiment of 0.63, 0.8 and 0.85 (Beck et al., 2020) (Fig. 2B). We provided our model with a force trace based on the predicted SOL force in Beck et al. (2020). Our model used an *l*_0_=0*.*0386 m based on data provided in Beck et al. (2020) and *F*_0_=3100 N similarly calculated using PCSA values from Handsfield et al. (2014). Here, we aimed to account for the energy cost owing only to the SOL muscle, so we scaled the input force and the experimental energetic cost assuming that force contributions to the total torque from each of the muscles was based on the relative PCSA of the triceps surae muscles [the medial (MG) and lateral (LG) gastronemii and the SOL]. Here, we neglect the possibility of increased activation related to the size of the muscle (Hug et al., 2015), as there tends to be large inter-subject variability (Hamard et al., 2022; Crouzier et al., 2018). Although Beck et al. (2020) suggested that the majority of the energetic cost is due to the SOL, they reported large activation levels in the LG [e.g. low duty high torque condition: peak LG activation ≈35 mV, peak SOL activation ≈45 mV (Beck et al., 2020), and did not measure EMG in the MG]. Because of this, we made the assumption that the LG and MG will also have contributions to the energetic cost (Crouzier et al., 2018). Using PCSA values from Handsfield et al. (2014) (124 cm^2^ SOL, 50 cm^2^ MG and 23 cm^2^ LG), we can scale the total energy by the SOL contribution to the total PCSA (60%). The width of the force–length relationship from Dick et al. (2017) was decreased to achieve a better comparison with the Beck et al. (2022) results; this was done for both the B2020 and the B2022 simulations. Tendon stiffness (*k*=15*.*5×10^4^ N m^−1^) was set to approximate the maximal experimental fascicle strain (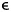
_max_≈0*.*05 to 0*.*1). The parameters for the energetic rates and force–velocity parameters were chosen based on slow fibres given the composition of fibres within the human SOL is predominantly slow (Feiereisen et al., 1997).

### Beck et al. (2022) simulation

Beck et al. (2022) found an increase in energetic cost when muscle operates at shorter fascicle lengths. Similarly to the B2020 simulations, we scaled the input forces and experimental energetic cost. We used an input force based on the experimental condition provided in Beck et al. (2022) (see Fig. 2C). Here, plantarflexion contractions were performed with the knee angle at 50 deg to isolate contributions from the SOL (Beck et al., 2022). In the experiments, the SOL fascicle length in the resting (inactive) state was 0.0415 m at an angle of 90 deg and 0.035 m at an angle of 120 deg. Our modelling approach attempted to replicate changes in fascicle length owing to changes in joint angles. The fascicle length change was incorporated into the calculation of contractile element strain,
(8)

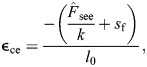
where *s*_f_ is the shift in the fascicle length (in m) and *k* is the normalized tendon stiffness (in m^−1^). To capture the three conditions, we used fascicle shifts *s*_f_ of 0, 0*.*1*l*_0_ and 0*.*2*l*_0_. Tendon stiffness (*k*=15*.*5×10^4^ N m^−1^) was set to maintain maximum strain 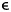
_max_≈0*.*05 from the resting length for a given ankle angle. Parameters for the energetic rates and the force–velocity properties were chosen to be the same as the B2020 simulations.

## RESULTS

### van der Zee and Kuo (2021) simulation

**Fig. 3.**
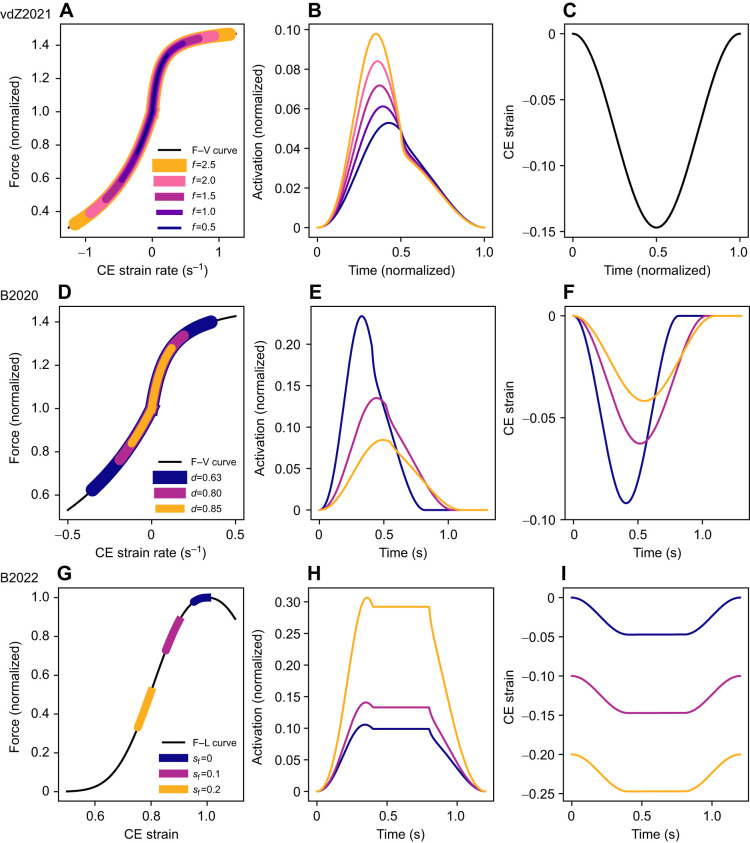
**Computed energetic costs for the** **van der Zee and Kuo (2021****) and** **Beck et al. (****2020,** **2022****) simulations.** (A–C) In the vdZ2021 simulations, faster cycle frequencies (*f*) resulted in traversals of larger regions of the force–velocity relationship (A) and higher activation levels (B), while the contractile element (CE) strain was kept constant (C). (D–F) In the B2020 simulations, lower duty cycles (*d*) and increased force resulted in traversals of larger regions of the force–velocity relationship (D), higher activations (E) and larger CE strains (F). (G–I) In the B2022 simulations, larger shifts in resting fascicle lengths (*s*_f_) resulted in shifts to the descending limb of the force–length relationship (G), higher activations (H) and similar relative strains in fascicle lengths with different resting strains (I).

**Fig. 4.**
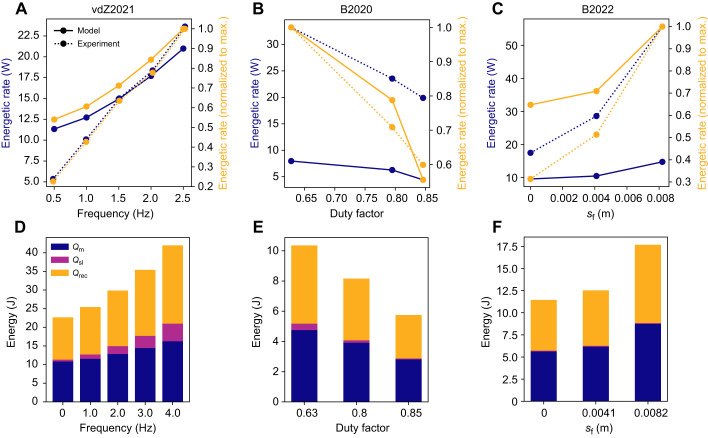
**Computed energetic costs for the** **van der Zee and Kuo (2021****) and** **Beck et al. (****2020,** **2022****) simulations.** (A,D) vdZ2021, (B,E) B2020 and (C,F) B2022. (A–C) The energetic cost increased with faster contraction rate (A), lower duty factors (B) and longer fascicle lengths (C). The relative changes in energetic cost with respect to the maximum energetic rates (yellow lines) demonstrate model the model’s ability to follow trends in the experimental data (*r*^2^=0.61, 0.91 and 0.39 for vdZ2021, B2020 and B2022, respectively). The *r*^2^ was calculated using the normalized metabolic rate data. The relative errors *e*_rel_ between the simulation and experimental data were 0.20, 3.10 and 2.23, respectively. (D–F) Increases in relative heat production over the contraction cycle from the shortening and lengthening heat component (*Q*_sl_) increased in the vdZ2021 (D) and B2020 (E) simulations with respect to the maintenance (*Q*_m_) and recovery heat rates (*Q*_rec_), while the relative heat production remained the same for the B2022 (F) simulations.

Our model predicted that as the frequency of contraction increased, the contractile element of the model traversed a larger region of the force–velocity relationship owing to the greater strain rates at higher contraction frequencies (Fig. 3A). This resulted in a higher muscle activation required to maintain the same force over the contraction cycle (Fig. 3B). Here we observed an increase in activation of 85% from 0.5 to 2.5 Hz, which is similar to the 75% increase measured experimentally (van der Zee and Kuo, 2021). van der Zee and Kuo (2021) found an increased energetic cost of 18.15 W (for one leg) when the contraction frequency increased from 0.5 to 2.5 Hz. Despite a qualitatively similar trend to the experimental results (*r*^2^=0*.*61 using normalized metabolic rates in Fig. 4A), our model predicted a smaller difference in energetic cost of 8.83 W between conditions, which corresponds to a relative error of 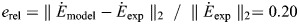 (Fig. 4A, dotted line). As the contraction frequency increased, the higher activation levels resulted in an increase in the maintenance heat, whereas the faster shortening rates resulted in larger contributions from the shortening heat (Fig. 4D). The model was able to predict similar trends in metabolic cost, but was not able to capture the energetic cost at low cycle frequencies.

### Beck et al. (2020) simulation

With a decrease in duty cycle, we observed an increase in the maximum strain rate over the contraction cycle (0.12 to 0.35 s^−1^), which corresponded to a larger region of the force–velocity relationship traversed in the model predictions (Fig. 3D). The increased strain rates resulted in an increase in activation level (from 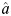
≈0*.*08 to 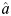
≈0*.*23) to maintain a given force (Fig. 3E). Beck et al. (2020) did not report the normalized activation levels, but when comparing the relative change in activation, the model displayed a 150% increase in 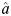 and experimentally there was an increase of ∼75%. The peak contractile element strains over a cycle predicted by the model were 0.091, 0.063 and 0.042 for duty cycles of 0.63, 0.80 and 0.85, respectively, whereas the respective experimental peak muscle fibre strains were 0.098, 0.081 and 0.056 (Fig. 3F). The model under-predicted the energetic cost from these experiments, as it only reached ∼14% of the scaled experimental values (*e*_rel_=3*.*10; Fig. 4B); however, when normalized to the peak energetic rate, we found similar qualitative trends in the energetic cost across duty cycles (*r*^2^=0*.*91 using normalized metabolic rates in Fig. 4B). There were increases in both the maintenance and shortening heat contributions to the total energy use, but higher strain rates led to a larger proportion of the total energy contributed by the shortening heat (Fig. 4E), consistent with the vdZ2021 simulations. The model demonstrated the ability follow trends in the energetics with changes to duty cycle, but under-predicted the magnitude of the energetic cost.

### Beck et al. (2022) simulation

The B2022 simulations investigated the effect of muscle fascicle operating length on energetic cost, and found that with increasing contractile element strain (modelling decreased fascicle lengths) there was a leftward shift in operating range on the force–length relationship (Fig. 3G), which led to large increases in muscle activation. To produce an equivalent peak force, the peak normalized activation increased from 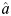
=0*.*105 to 0*.*309 when resting fibre strain factor was increased from *s*_f_=0 to 0.2*l*_0_ (Fig. 3H). This is approximately the same relative increase in activation levels, but smaller magnitudes than observed experimentally (0.25 to 0.77 increase in activation; Beck et al., 2022). The peak fascicle strains predicted by the model were 0.047, 0.147 and 0.247 for *s*_f_=0, 4 and 8 mm, respectively (Fig. 3I), whereas the experimental peak fascicle strains were 0.05, 0.15 and 0.22 for initial shifts in fascicle length of 0, 2.9 and 6 mm, respectively (Beck et al., 2022). The peak strain rates of 0.18 s^−1^ observed was the same between conditions, and ∼30% smaller than the experimental results (see Beck et al., 2022). The energetic cost of the predicted contractions showed a trend similar to the experimental data (*r*^2^=0*.*40 using normalized metabolic rates in Fig. 4C) but was much smaller in magnitude (*e*_rel_=2*.*23). Shorter fascicle lengths resulted in an ∼54% increase in energetic rate, whereas experimental measures showed an ∼200% increase (Fig. 4C). The difference in activation level between conditions was largely responsible for alterations in energetic cost, as the shortening heat contribution was unchanged under varying *s*_f_ (Fig. 4F). The model demonstrated the ability follow trends in the energetics with changes to resting lengths, but under-predicted the magnitude of the energetic cost.

### Sensitivity analysis

**Fig. 5.**
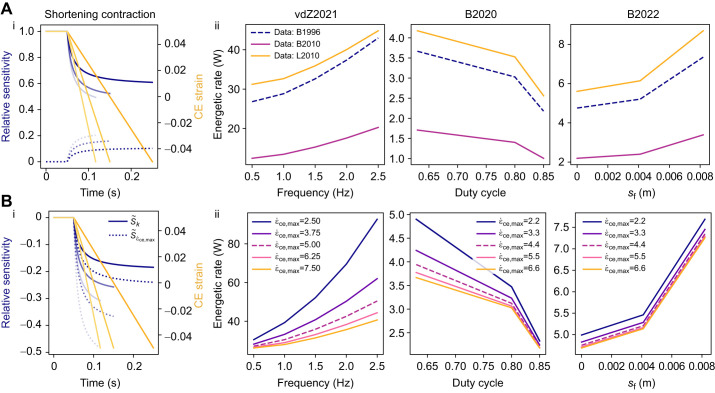
**Model sensitivities during shortening contractions in the** **van der Zee and Kuo (2021****) and** **Beck et al. (****2020,** **2022****) simulation protocols.** (A) Sensitivity to energetic parameters. (Ai) Relative sensitivity to the energetics parameters, *r*_1_ (solid lines) and *r*_2_ (dotted lines). (Aii) Sensitivity to the energetic data used to determine model parameters. These data were taken from Barclay (1996) (B1996), Barclay et al. (2010) (B2010) and Lichtwark and Barclay (2010) (L2010). (B) Sensitivity in the force–velocity parameters. (Bi) Relative model sensitivity to the force–velocity curvature, *κ* (solid lines) and the maximum strain rate, 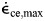 (dotted lines). (Bii) 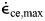 was varied by ±25% and ±50%. Dashed lines represent the base parameter set used for previous experiments. The relative sensitivities were calculated as the ratio of a percent change in the parameter to a percent change in the predicted energy use. 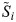 denotes the relative sensitivity with respect to parameter *i*. Shortening contractions were simulated at shortening rates of 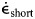
=0*.*5, 1.0 and 1.5 s^−1^, which corresponds to fading colour on the plots (dark to light), and using slow-type fibre properties.

To understand the influence of specific modelling parameters on the muscle model energy predictions, we performed a sensitivity analysis (Fig. 5). We chose to perform the sensitivity analysis on the parameters that demonstrate large experimental variation and are challenging to experimentally measure: the energetic parameters *r*_1_ and *r*_2_, and the force–velocity constants κ and 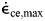. This analysis consisted of (i) a relative sensitivity analysis (Fig. 5Ai,Bi) during a shortening contraction whereby the muscle was activated to 100% activation and then allowed to shorten at fixed velocities of 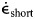
=0*.*5, 1.0 and 1.5 s^−1^ over the plateau of the force–length relationship, and (ii) a sensitivity analysis (Fig. 5Aii,Bii) whereby we investigated the sensitivity of the predicted energy rate under the vdZ2021, B2020 and B2022 simulation protocols to the input parameters. The relative sensitivities correspond to a ratio between a percent change in the parameter and a percent change in the predicted energy use; in particular, 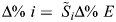, where Δ% is the percent change in parameter *i*, 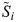 is the relative sensitivity to *i* and Δ%*E* is the percent change in the energy.

The sensitivity analysis on the energetic parameters demonstrated that the model was most sensitive to the maintenance heat parameter, *r*_1_, during the isometric contraction with a relative sensitivity 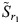
=1*.*0 while 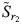
=0*.*0 (Fig. 5Ai). During the shortening period, energy use became less sensitive to *r*_1_ and the sensitivity to the shortening heat rate parameter, *r*_2_, increased, while still maintaining higher sensitivity to *r*_1_ (Fig. 5Ai). Given the high sensitivity to the energetic parameters, we examined how using different data to inform these parameters would influence the outputs in the vdZ2021, B2020 and B2022 simulation protocols. In addition to the Barclay (1996) parameter values given in Table 2, the energetics parameters were determined based on the data from Barclay et al. (2010) and Lichtwark and Barclay (2010). This effectively corresponds to a range in *r*_1_ from 0.38 to 0.74 s^−1^ and a range in *r*_2_ from 0.09 to 0.23, demonstrating high variability in the data used to derive energetic model parameters. Considering these datasets, we found that the predicted energetic rates can vary by ∼150% depending on the experimental condition (Fig. 5Aii). The datasets considered here measured heat rates in slow-twitch SOL muscle during fibre-bundle experiments (for the methods used to determine parameter values, see the Supplementary Materials and Methods).

The sensitivity analysis on the force–velocity relationship curvature, κ, and maximum shortening rate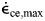 demonstrated that during shortening the model energy use was more sensitive to 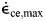 compared with κ (Fig. 5Bi). Note that the negative relative sensitivity values indicate a percent change in the variable will result in the corresponding decrease in the energetic cost. As expected, during an isometric contraction (with no tendon), there was no influence of κ and 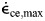 on energy use (Fig. 5Bi). Given the model was more sensitive to 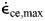, we examined the sensitivity during the vdZ2021, B2020 and B2022 simulations by varying 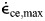 by ±25 and ±50%. Fig. 5Bii demonstrates the strong sensitivity on 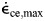 at the high contraction rates observed in vdZ2021 and B2020 protocols. B2020 simulations were less sensitive to 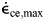 with no change in the magnitude of the sensitivity between conditions, which is expected based on limited changes to the shortening rates (see Fig. 3I).

## DISCUSSION

This study investigated the ability of a physiologically based muscle model (modified from Lichtwark and Wilson, 2005, 2007) to capture the energetics of *in vivo* skeletal muscle contraction under a range of mechanical conditions (van der Zee and Kuo, 2021; Beck et al., 2020, 2022). Specifically, we explored the relationship between muscle mechanics and energetic cost, and demonstrated that these models are sufficient to qualitatively capture and explain these relationships. The model utilized in this study is part of a class of phenomenological models that predicts energetics based on the mechanical state of the muscle. Previously, this class of models has been shown to both accurately predict (Tsianos and MacFadden, 2016) and under-predict energetic cost (Lentz-Nielsen et al., 2023) depending on the available data and experimental condition. The inconsistencies in energetic predictions could be due to the poor ability of the model to predict energy use with changes to muscle mechanical state, which warrants an investigation of the ability of physiologically based models to capture the relationship between the muscle mechanics governing muscle contraction and energetic cost. In many cases, physiologically based models have been applied to study changes in net whole-body energetics during complex locomotor tasks (Miller, 2014; Koelewijn et al., 2019); however, even during simple single joint movements, whereby the neuromechanical contributions of an individual muscle or single group of muscles can be interpreted, model predictions of *in vivo* energetics are not completely understood. Our results demonstrate that physiologically based models are able to relate the changes in muscle mechanical state to the general patterns in energetic rates measured experimentally, but the absolute values tend to vary considerably from measures of muscle energy use from indirect calorimetry. The experimentally informed modelling framework developed here provides rich insights into muscle energetics, but the discrepancies between model predictions and experimental measures warrants further exploration.

### Energetic rate predictions

In the vdZ2021 simulations, the energetic rates calculated by the model were able to predict the trends in the experimental data, but the magnitude differed between the model predictions and the experiments. van der Zee and Kuo (2021) found that the model by Uchida et al. (2016) was not able to capture the trend in the energetic cost with contraction frequency, only predicting ∼7% of the change in energetic cost that was measured in their experiments. This under-prediction lead to the suggestion that other mechanisms are required to explain the energetic cost associated with the rate of force development. Here, we found that the Lichtwark and Wilson (2005, 2007) model with experimentally based parameters was able to capture ∼50% of the experimentally measured change in the energetic cost, but over-predicted energetic cost values at low cycle frequencies and under-predicted at high frequencies. One possible explanation could be errors in the force–velocity relationship leading to errors in the predicted activation at high and low cycle frequencies. There are a number of other possible reasons for the discrepancy in absolute energetic rate, which could range from assumptions about the sources of energy in the experiment (e.g. co-contraction in other muscles at fast cycle frequencies and due to stabilization) to inadequate scaling of parameters to whole-muscle energetics. Further, although the model accounts for the recovery heat rates by assuming a constant overall efficiency at the steady state conditions in the experimental studies (van der Zee and Kuo, 2021; Beck et al., 2020, 2022), these rates are not sufficient to accurately match the magnitude of the energetic costs in the Beck et al. (2020, 2022) experiments. A more detailed, time-dependent model of the recovery processes may provide more insight (Morton, 1986), but will not likely lead to substantial increases in magnitude of energetic cost. As shown in Tsianos and MacFadden (2016), accounting for additional processes through an additional constant energetic rate helps to match the magnitude of the metabolic cost at the whole-body level, but the exact constant will be dependent on the task and the aforementioned factors.

The values of energetic rate measured in the single joint studies by Beck et al. (2020, 2022) are potentially high considering the simplicity of the task (e.g. metabolic rate is 20% of net walking metabolic rate; Umberger, 2010). There are a number of possible explanations, particularly relating to the high chance of metabolic energy consumption occurring due to other muscle contractions and/or physiological processes (e.g. breathing). For example, there is likely a high amount of co-contraction occurring in other lower limb muscles, such as the more proximal and larger hamstrings and quadriceps muscles, or in the postural muscles that stabilized the body. Beck et al. (2020) did show that there was a large amount of co-activation in the antagonist tibialis anterior (TA), which was not accounted for in our scaling of the experimental energetic rates. To verify that co-contraction was occurring in the other lower limb muscles, we conducted a pilot experiment in one participant (see the Supplementary Materials and Methods), where we replicated the Beck et al. (2020) protocol, but with sEMG measurements on the ankle plantar flexors, ankle dorsiflexors, knee extensors and knee flexors. We found that there was co-contraction occurring in the TA and the proximal leg muscles (vastus lateralis, vastus medialis, semitendinosis and biceps femoris), and given the larger volumes of the proximal leg muscles, this likely has a substantial contribution on the whole-body energetic cost. Antagonist muscle co-contraction is a problem in musculoskeletal model parameter estimation, even at the single joint level, limiting our ability to accurately capture energetics (Hasson et al., 2011). However, our models do capture the general trends in the data with the effects of muscle activation dominating the changes in energetic cost with both changes in duty cycle and muscle fascicle length.

### Mechanics inform energetics

Together, our modelling results demonstrate that the mechanics of muscle contraction is the primary driver of muscle energy use during the range of tasks explored within. Across the three studies, we identified two primary mechanisms that contribute to the observed changes in energetic cost with changes in (i) cycle frequency (van der Zee and Kuo, 2021), (ii) duty cycle (Beck et al., 2020) and (iii) fascicle length (Beck et al., 2022). In the first two studies, the changes to contraction frequency and duty cycle resulted in an increased strain rate, which, as seen in Results sections on van der Zee and Kuo, 2021 and Beck et al., 2020 simulations, required higher activation levels to maintain the same level of force (Aeles et al., 2022). The increased strain rate resulted in higher activation, which resulted in greater maintenance heat rates, along with larger relative contributions from the shortening and lengthening heat rates. The importance of the shortening rate on energetics is demonstrated in the model through the high sensitivity of the energetic rates to the maximum shortening rate of the muscle (Fig. 5). The exact shape of the force–velocity relationships often varies and is not known for the majority of muscles, which adds to the uncertainty in many modelling studies. Increases in shortening heat owing to faster shortening velocities, also known as the Fenn effect (Fenn, 1923), have been demonstrated to occur *in vivo* (Ortega et al., 2015). The second mechanism is the decreased fascicle lengths, which required higher activation levels owing to the leftward shift in the muscle's operating region on the force–length relationship (see Fig. 3G). In the B2022 simulations, we found no change in the distribution between the maintenance and shortening heat rates, which is to be expected given shifts in the fascicle length only resulted in changes to the operating lengths of the muscle and not the strain rates of the fascicles.

### Experimental data informing models

In addition to investigating the main drivers behind energetic cost, the other purposes of this study were to (i) investigate the role of both input data on model predictions and (ii) understand *in vivo* experimental data (normalized activation levels, maximum isometric force measures, etc.) needed to obtain accurate predictions of isolated muscle or groups of muscle energetics. We investigated the influence of input heat rate data obtained via isolated fibre bundle experiments from three sources (Barclay, 1996; Barclay et al., 2010; Lichtwark and Barclay, 2010), and illustrated that the range in experimental inputs can have a substantial impact on overall energetic cost. Fibre bundle thermopile experiments are highly complex, and only a few select groups engage in such endeavours (for a review, see Woledge et al., 1985; Barclay and Curtin, 2023); hence, caution should be exercised when selecting data to inform model parameters. Further, the energetic rates may differ in human muscle relative to mouse muscle; for example, different myosin isoforms may lead to different rates of ATP splitting (Reggiani et al., 1997) or Ca^2+^ binding proteins (e.g. parvalbumin; Celio and Heizmann, 1982) may alter the energetic rates (Barclay and Curtin, 2023), but the relative difference in determining human muscle parameters is not clear. The sensitivity analysis (Fig. 5) demonstrates the potential role these differences may play. Some studies have investigated heat released in humans *in vivo* (Bolstad and Ersland, 1978), but this can only be done during isometric experiments. They found that for the SOL, the heat rate released during a maximum isometric contraction was ≈16 W, which is smaller than the ≈50 W predicted by our model. The difference potentially owing to the loss of heat to the environment through the skin, other tissues or blood flow, which is difficult to limit. To avoid consequences due to losses in heat to the environment during thermopile experiments and the effects of co-contraction in whole-body indirect calorimetry measures, functional phosphorous magnetic resonance spectroscopy (P-MRS) can be used (Boska, 1994), which provides a measure of the phosphocreatine breakdown indicative of ATP hydrolysis (e.g. Barclay and Curtin, 2023) providing more reliable *in vivo* data to inform energetic models. P-MRS has been validated with mathematical models (Haeufle et al., 2020; Callahan et al., 2013); however, it is restricted to isometric contractions and could not be used in the investigation of more dynamic conditions. The sensitivity analysis of the model (Fig. 5) demonstrated the role not only of the energetic parameters on the energetic cost, but also of the force–velocity relationship parameters. There is a high level of dependence, especially during shortening contractions, as the location of the muscle on the force–velocity curve will drive changes in muscle activation level.

In addition to the intrinsic muscle parameters that can be obtained from isolated fibre-bundle experiments, there are data that could be non-invasively measured during *in vivo* human experiments that could improve the modelling predictions. One key contributing factor is co-contraction, which can drive changes in both the magnitude and the qualitative trends in experimentally measured energetic costs. To limit this, researchers could provide biofeedback on co-contraction levels to the participant or focus on single muscle joints (e.g. abduction of the second digit in the hand is done solely by the first dorsal interosseous). Another variable is the maximum isometric force produced by the muscle. The maximal torque at the joint level needs to be reported as this will alter both the mechanics, through the prediction of muscle activation, and the scaling of the energetic rates. Reporting muscle activation data that is normalized to maximum voluntary contractions also provides a key measure to compare with model predictions. One aspect that was not investigated in this study, but will likely play a role in energetic cost predictions at higher intensities, is the role of motor unit recruitment. Given the large difference in energetic rates between slow- and fast-type muscle fibres (see Table 2), it is likely that the motor unit recruitment strategy will substantially influence energetic cost (Swinnen et al., 2024).

### Limitations and future directions

The model implemented and tested here has limitations that could be improved in future work. First, the experimental data used as input for the model (e.g. maximum isometric force, optimal fascicle length, etc.) were typically based on data averaged across individuals, in some cases from different studies, and so the parameters used may not represent the experimental sample or an individual subject. Further, there can be large variations in energetic rates [Beck et al. (2020) found metabolic cost could vary by 100 W between participants in some conditions]; thus, this could likely be improved upon by predicting energetic cost on a subject-specific basis. Second, this study used a simplified Hill-type model to describe the mechanics of muscle contraction, which fails to account for effects from the architecture of muscle, muscle mass and detailed tendon mechanics. We found that shortening rate plays an important role in muscle energetics; however, our results are limited to a Hill-type model neglecting architectural factors such as variable pennation angles and gearing (Dick and Wakeling, 2017, 2018), which could play a role in altering energetic costs. To better capture multi-dimensional muscle shape changes (e.g. Kelp et al., 2023), a three-dimensional model of muscle (e.g. Blemker and Delp, 2005; Dick and Wakeling, 2018; Almonacid et al., 2024) would allow us to account for not only the effects of architecture on the muscle fascicle shortening but also the energy required to deform the muscle itself (Wakeling et al., 2020; Ross et al., 2021; Ross and Wakeling, 2021). Another aspect that has been simplified in the model is the use of a constant tendon stiffness. Tendon stiffness has been shown to play a role in the energetics of muscle (Lichtwark and Wilson, 2007), and a more accurate model that accounts for nonlinear tendon properties could alter the energetic rates predicted by the model.

Future studies could also include a motor unit recruitment model (e.g. Fuglevand et al., 1993; Caillet et al., 2022) to better capture the energetic cost at higher levels of activation. Previous models (Callahan et al., 2013) have accounted for detailed recruitment characteristics, but have not been used to investigate dynamic contractions where changes to the recruitment strategies and the current energetics models tend to underperform. In the simulations here, the contractions were either at very low activation levels (vdZ2021) or in muscles with very low proportions of fast fibres (B2020 and B2022), thus a recruitment model was not implemented for this study. In addition to the recruitment model, accounting for excitation–activation coupling (e.g. Mayfield et al., 2023) may provide more realistic energetic costs in repeated contractions, where the Ca^2+^ concentrations may not return to a baseline level over multiple contractions. Models that are more biophysically motivated, which include detailed activation dynamics, have been developed for whole-muscle simulations (Ma and Zahalak, 1991), but are not as widely utilized in whole-body simulations of movement.

### Conclusions

The physiologically based muscle model used in this study was able to capture the trends in muscle energy use observed during *in vivo* tasks across a range of contraction conditions (van der Zee and Kuo, 2021; Beck et al., 2020, 2022). The model was unable to accurately capture the absolute value of the experimental energetic rates, which could be due to many factors, such as co-contraction of other muscles, estimated maximum isometric force values, or motor unit recruitment. Accurate reporting of the aforementioned factors could help isolate individual muscle contributions to whole-body energetic rates. Our results indicate that this model can be improved upon to better capture changes in energetic rate, which could include a motor unit recruitment model or a more detailed mechanical model. To further our understanding of *in vivo* muscle energetics, studies designed to inform specific energetic model properties (e.g. recovery energetic rates or *in vivo* motor unit recruitment) will provide invaluable data to verify the capabilities of existing energetics models and solidify current understanding. This study details a modelling framework that utilizes *in vivo* experimental measures to capture muscle energetics and demonstrates that the muscle mechanical state is a key determinant in predictions of energy use.

## Supplementary Material

10.1242/jexbio.249966_sup1 Supplementary information
