# Supplementary material for: Using physiologically based models to predict in vivo skeletal muscle energetics
Source: J Exp Biol. 2025 Mar 31;228(7):jeb249966. doi: 10.1242/jeb.249966 (PMC11993265; doi:10.1242/jeb.249966)
Supplement: Supplementary information [file jexbio-228-249966-s1.pdf]

Journal of Experimental Biology • Supplementary information

Supplementary Materials and Methods

1.1 Determining model parameters

In this study we used data from experiments on isolated mammalian fibre bundle experiments. In particular, we use data from Barclay [1996], Barclay et al. [2010], and Lichtwark and Barclay [2010]. Aside from Lichtwark and Barclay [2010], these experiments are not performed at body temperature, and thus needed to be scaled using appropriate Q10 factors. Further, we normalized all of our parameters by $F_0\, l_0$. This section details the scaling performed to the experimental data, and the values obtained for the heat rate constants r_1 and r_2. The values for the parameters are given in Table S1. The derivation of the parameter for the [Barclay, 1996] and [Barclay et al., 2010] data sets are given in subsubsection 1.1.1 and subsubsection 1.1.2, respectively.

Table S1. Energetic parameters obtained from Barclay [1996], Barclay et al. [2010], Lichtwark and Barclay [2010]. Lichtwark and Barclay [2010] only performed experiments on the SOL muscle.

	Barclay 1996	Barclay 2010	Lichtwark 2010
r_1, SOL (s^{-1})	0.62	0.38	0.74
r_1, EDL (s^{-1})	2.79	1.13	N/A
r_2, SOL ()	0.23	0.11	0.09
r_2, EDL ()	0.70	0.07	N/A

1.1.1 Barclay 1996 Parameter Scaling

To obtain the parameters from the [Barclay, 1996] dataset, we scaled the energetic rates from mW (g dry mass)$^{-1}$ to s^{-1}. The maximum isometric force measured experimentally, $F_{0,exp}$, was reported in units of Nm (g dry mass)$^{-1}$, so to obtain units in N, we multiplied the experimental values by the dry mass, m_d, and divided by the fascicle length, l_0. The scaled maximum isometric stress $\tilde{F}_0$ is then given by

$$\tilde{F}_0 = F_{0,exp} \frac{m_d}{l_0}. \tag{S1}$$

For these experiments, we scaled for temperature assuming a Q10 value of 4 for the heat rates [Rall and Woledge, 1990], 3.5 for the power, and a value of 2 [Ranatunga, 1998] for the maximum shortening rate. The adjusted energetic rates (power and heat rates) can be computed using

$$\dot{Q}_{exp,scaled} = \dot{Q}_{exp} \frac{m\, Q_{10,Heat}}{\tilde{F}_0\, l_0}, \tag{S2}$$

$$P_{exp,scaled} = P_{exp} \frac{m\, Q_{10,Power}}{\tilde{F}_0\, l_0}. \tag{S3}$$

In these experiments the scaling parameters are muscle specific for the soleus (slow-type muscle fibre) and extensor digitorum longus (fast-type muscle fibre), and are given in Table S2. The parameters r_1 and r_2 can then be obtained through a linear fit to the experimental data, so we get $\dot{Q} = r_2\, \dot{\epsilon}_{ce} + r_1$.

1.1.2 Barclay et al. 2010 scaling

Barclay et al. [2010] reports energetic data for the soleus (SOL) and extensor digitorum longus (EDL) muscles during shortening contractions. The data is linearly extrapolated to 35°C, since data is given at

Table S2. **Parameters used in the scaling of the heat rates from the Barclay [1996] and Barclay et al. [2010] dataset.** The $\dot{\epsilon}_{max}$ values are before they have been scaled to account for temperature effects. κ is the curvature in the force-velocity relationship (see Dick et al. [2017]). Note that fibre lengths were not reported in Barclay et al. [2010], so we used values from Barclay [1996].

Parameter	Value	Source
Barclay 1996 Dataset		
$Q_{10,Heat}$	4	Value from Rall and Woledge [1990]
$Q_{10,Power}$	3.5	Value from Rall and Woledge [1990]
$Q_{10,\dot{\epsilon}_{max}}$	2	Value from Ranatunga [1998]
$\dot{\epsilon}_{max,SOL}$	$3.15\ s^{-1}$	Value from Barclay [1996]
κ_{SOL}	0.20	Value from Barclay [1996]
$F_{0,SOL}$	$90.6\times10^{-3}\ N$	Value from Barclay [1996]
m_{SOL}	$0.87\ \times10^{-3}\ g$	Value from Barclay [1996]
$l_{0,SOL}$	$9.7\times10^{-3}\ m$	Value from Barclay [1996]
$\dot{\epsilon}_{max,EDL}$	$5.61\ s^{-1}$	Value from Barclay [1996]
κ_{EDL}	0.37	Value from Barclay [1996]
$F_{0,EDL}$	$136.5\times10^{-3}\ N$	Value from Barclay [1996]
m_{EDL}	$1.04\ \times10^{-3}\ g$	Value from Barclay [1996]
$l_{0,EDL}$	$9.75\times10^{-3}\ m$	Value from Barclay [1996]
Barclay et al. 2010 Dataset		
$\sigma_{0,SOL}$	$20.6\times10^{5}\ N\,m^{-2}$	Average value from Barclay et al. [2010]
m_{SOL}	$3.4\times10^{-3}\ g$	Value from Barclay et al. [2010]
$l_{0,SOL}$	$9.7\times10^{-3}\ m$	Value from Barclay [1996]
$\sigma_{0,EDL}$	$22.6\times10^{5}\ N\,m^{-2}$	Average value from Barclay et al. [2010]
m_{EDL}	$3.9\ \times10^{-3}\ g$	Value from Barclay et al. [2010]
$l_{0,EDL}$	$9.75\times10^{-3}\ m$	Value from Barclay [1996]
ρ	$1.06\ \times10^{6}\ g\,m^{-3}$	Density of muscle

temperatures of 25, 25, and 30 $°C$. The resulting parameters are then scaled to units of s^{-1} similarly to the Barclay [1996] dataset using the parameters given in Table S2. Since Barclay et al. [2010] report the maximum isometric stress, the maximum isometric force was computed using

$$F_0 = CSA\,\sigma_0, \tag{S4}$$

where CSA is computed as

$$CSA = \frac{m}{\rho\, l_0}. \tag{S5}$$

The scaling of the parameters is then given by

$$r_i = \frac{\tilde{r}_i}{F_0\, l_0}, \tag{S6}$$

where $\tilde{r}_i$ is the corresponding heat rate parameters ($i = (1, 2)$) in $W\,g^{-1}$.

Maintenance heat rate parameter, r_1**:** Barclay et al. [2010] reports the isometric heat rates which corresponds to the r_1 parameter in our model. Data is reported at temperatures of 20, 25, and 30 $°C$, so the parameter values at body temperature $\approx 35°C$ are linearly extrapolated from the data (Figure S1).

Shortening heat rate parameter, r_2**:** Data for the enthalpy and power of shortening contractions are shown in Barclay et al. [2010] Figure 2. We can then calculate the heat rates by subtracting the power

Journal of Experimental Biology • Supplementary information

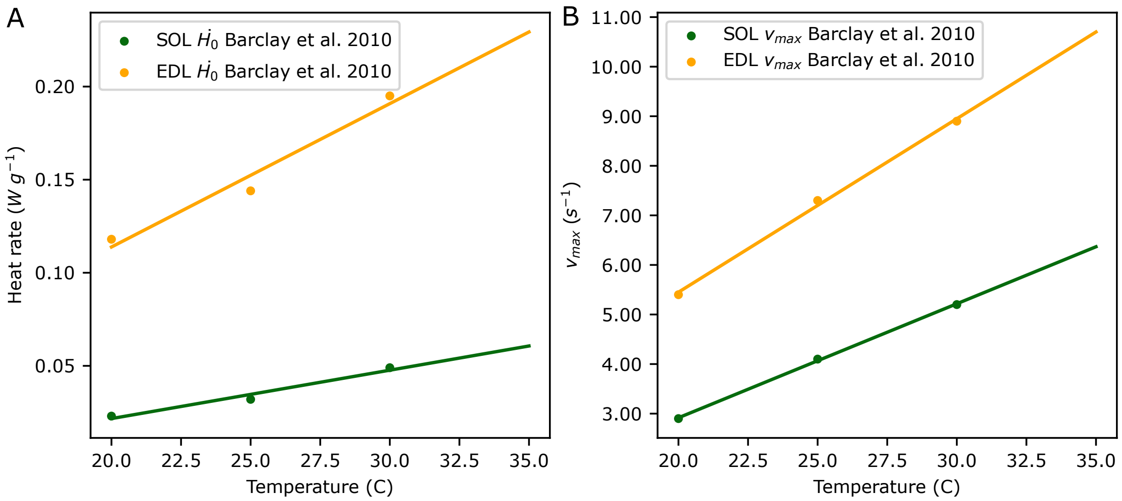

Fig. S1. Linear extrapolation of the isometric heat rate ($\dot{H}_0$, $W\,g^{-1}$) and maximum shorten-ing velocity (v_{max}, s^{-1}) to $35°C$. Data from Barclay et al. [2010]

from the enthalpy (Figure S2 A,B). The parameter r_2 can then be determine by the slope of the heat rate line, assuming a linear relationship between heat and shortening rate (Figure S2 C,D). The parameter used in this study can be extracted from the linear extrapolation to $35°C$ (Figure S2, E,F).

Journal of Experimental Biology • Supplementary information

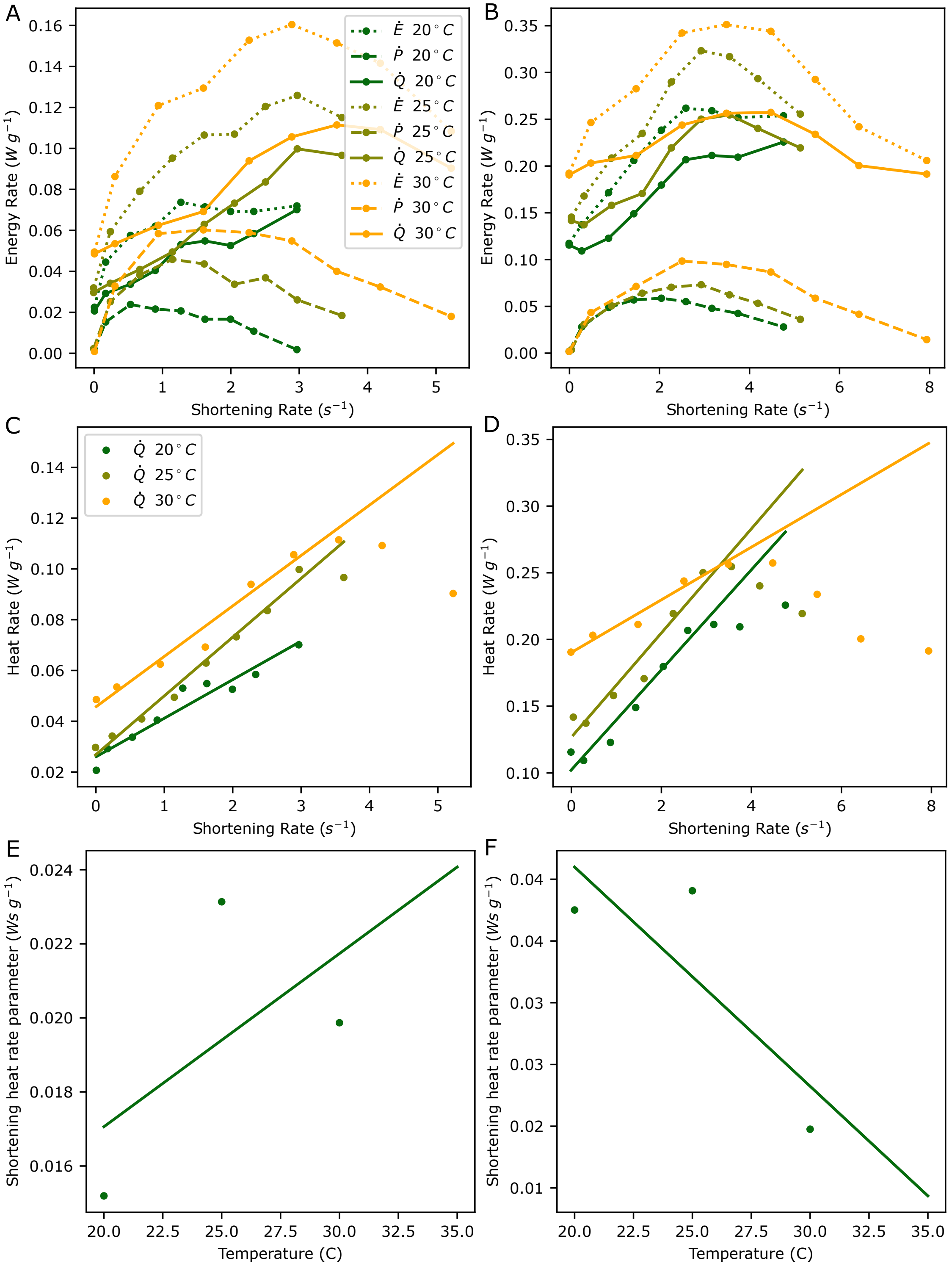

Fig. S2. Determining the shortening heat rate parameter from Barclay et al. [2010] dataset. Plots of the total enthalpy, heat rate, and power from Barclay et al. [2010] for the SOL (A) and EDL (B) at temperatures of 20, 25, and 30 °C. Fit of the heat rate data to obtain the shortening rate parameter (top) for the SOL (left) and EDL (right), which is given by the slope of the line. The parameter is then extrapolated to 35 °C (bottom).

Journal of Experimental Biology • Supplementary information

1.2 Parameters used in numerical experiments

Table S3. Parameters used in the computational experiments. vdZ2021, B2020, and B2022, cor-respond to the simulations used to replicate the van der Zee and Kuo [2021], Beck et al. [2020], and Beck et al. [2022] experiments, respectively. κ is the curvature in the intrinsic force-velocity relationship.

Parameter	vdZ2021	B2020	B2022
$\dot{\epsilon}_{ce,max}$	$5\,s^{-1}$	$4.4\ s^{-1}$	$4.4\ s^{-1}$
κ	0.17	0.17	0.17
F_0	4775 N	3100 N	3100 N
l_0	0.095 m	0.0386 m	0.041 m

Journal of Experimental Biology • Supplementary information

1.3 Sensitivity analysis

To investigate the sensitivity of the energetics model to the parameters r_1 and r_2, we can compute the relative sensitivities of the total energy. This can be done following the procedure from Rockenfeller et al. [2015] by calculating the sensitivity, S_ξ, to a given parameter ξ. Let $\dot{E}(t;\Sigma)$ be the energetic rate at time t with parameter set Σ. We can then define the sensitivity of the energy to parameter $\xi \in \Sigma$ as

$$S_\xi(t;\Sigma) = \frac{d}{d\xi}E(t;\Sigma). \tag{S7}$$

Since E is unknown, we can take the derivative with respect to time from both sides and we get

$$\dot{S}_\xi(t;\Sigma) = \frac{d^2}{dtd\xi}E = \frac{d}{d\xi}\dot{E}. \tag{S8}$$

We can then integrate with respect to time to get S_ξ. To understand the sensitivity of each parameter relative to the others, we can compute the relative sensitivity, $\tilde{S}_\xi$, as

$$\tilde{S}_\xi = S_\xi \frac{\xi}{E(t)}. \tag{S9}$$

The relative sensitivity gives the ratio between a percentage change in the parameter ξ and the energy E.

For our model we have $\Sigma = (r_1, r_2, \kappa, \dot{\epsilon}_{ce,max})$. We can compute the derivatives for S_{r_1} and S_{r_2}

$$\dot{S}_{r_1} = \frac{d}{dr_1}\dot{E} = \frac{d}{dr_1}\dot{Q}_m = \hat{a}\,(0.3 + 0.7\hat{F}_{ce,l}(\epsilon_{ce}))\begin{cases} 1 \text{ if } \dot{\epsilon}_{ce} < 0, \\ 0.3 + 0.7\,e^{-8\dot{\epsilon}_{ce}} \text{ if } \dot{\epsilon}_{ce} \geq 0, \end{cases} \tag{S10}$$

$$\dot{S}_{r_2} = \frac{d}{dr_2}E = \frac{d}{dr_2}\dot{Q}_{sl} = \hat{a}\,F_{ce,l}(\epsilon_{ce}))\begin{cases} -\dot{\epsilon}_{ce} \text{ if } \dot{\epsilon}_{ce} < 0, \\ 0 \text{ if } \dot{\epsilon}_{ce} \geq 0. \end{cases} \tag{S11}$$

The sensitivity to $\dot{\epsilon}_{ce,max}$ and κ depends on the mechanical model and the derivatives $\frac{d\hat{F}_{ce,v}}{d\dot{\epsilon}_{ce,max}}$ and $\frac{d\hat{F}_{ce,v}}{d\kappa}$. Using the intrinsic properties from Dick et al. [2017], we have

$$\frac{d\hat{F}_{ce,v}}{d\dot{\epsilon}_{ce,max}} = \begin{cases} \frac{-\dot{\epsilon}\,(1+\frac{1}{\kappa})}{(\dot{\epsilon}_{ce,max}-\frac{\dot{\epsilon}}{\kappa})^2}, \text{ if } \dot{\epsilon} < 0, \\ -0.5\,\frac{\dot{\epsilon}\,(1+\frac{7.56}{\kappa})}{(\dot{\epsilon}_{ce,max}+7.56\frac{\dot{\epsilon}}{\kappa})^2}, \text{ if } \dot{\epsilon} < 0, \end{cases} \tag{S12}$$

and

$$\frac{d\hat{F}_{ce,v}}{d\kappa} = \begin{cases} -\frac{1+\frac{\dot{\epsilon}}{\dot{\epsilon}_{ce,max}}}{\left(1-\frac{\dot{\epsilon}}{\dot{\epsilon}_{ce,max}\kappa}\right)^2}\frac{\dot{\epsilon}}{\dot{\epsilon}_{ce,max}\kappa^2}, \text{ if } \dot{\epsilon} < 0, \\ -0.5\frac{1-\frac{\dot{\epsilon}}{\dot{\epsilon}_{max}}}{\left(1+7.56\frac{\dot{\epsilon}}{\dot{\epsilon}_{ce,max}\kappa}\right)^2}\frac{7.56\dot{\epsilon}}{\dot{\epsilon}_{ce,max}\kappa^2}, \text{ if } \dot{\epsilon} \geq 0. \end{cases} \tag{S13}$$

The sensitivities can then be computed via chain rule

$$\dot{S}_{\dot{\epsilon}_{ce,max}} = \frac{d\dot{E}}{d\dot{\epsilon}_{ce,max}} = \frac{d\dot{Q}_m}{d\hat{a}}\frac{d\hat{a}}{d\dot{\epsilon}_{ce,max}} + \frac{d\dot{Q}_{sl}}{d\hat{a}}\frac{d\hat{a}}{d\dot{\epsilon}_{ce,max}}, \tag{S14}$$

or, expanding,

$$\dot{S}_{\dot{\epsilon}_{ce,max}} = \left[\frac{d\dot{Q}_m}{d\hat{a}} + \frac{d\dot{Q}_{sl}}{d\hat{a}}\right] \frac{-\hat{F}}{\hat{F}_{ce,l}\hat{F}^2_{ce,l}} \frac{d\hat{F}_{ce,v}}{d\dot{\epsilon}_{ce,max}}. \tag{S15}$$

Similarly for $\dot{S}_\kappa$ we get

$$\dot{S}_\kappa = \left[\frac{d\dot{Q}_m}{d\hat{a}} + \frac{d\dot{Q}_{sl}}{d\hat{a}}\right] \frac{-\hat{F}}{\hat{F}_{ce,l}\hat{F}^2_{ce,l}} \frac{d\hat{F}_{ce,v}}{d\kappa}. \tag{S16}$$

Journal of Experimental Biology • Supplementary information

1.4 Pilot experiments to examine co-contraction

The energetic cost predictions for the B2020 and B2021 studies are much higher than the predictions from the model. To understand the potential reason for these differences, we constructed a similar experimental setup to the Beck et al. [2020] study, with knee angle at 50° and ankle angle at 90°, and asked a participant to perform the high torque conditions to determine if we get a similar energetic cost. In addition to measuring ankle torque and energetic cost, we also measured EMG (Trigno Avanti, 2048 Hz, inter-electrode distance: 10 mm, Delsys Inc., USA) on the soleus (SOL), medial gastronemius (MG), lateral gastronemius (LG), tibialis anterior (TA), vastus lateralis (VL), vastus medialis (VM), semitendonosis (ST), and biceps femoris (BF). This allows for us to understand the co-activation that could be occurring, which would contribute to the energetic cost. Changes were also made to the setup of the experiment. Instead of the seated position from Beck et al. [2020], we used a prone position due to constraints on available equipment. We find similar energetic costs as Beck et al. [2020] despite the changes in the experimental setup. Beck et al. [2020] found metabolic rates of 33, 39, and 55 W for duty factors 0.8, 0.6, and 0.4, while we found corresponding energetic rates of 30, 35, and 53 W.

The EMG data were band-pass filtered through a 20-500Hz filter. The mean was subtracted then the data was rectified. Finally, the data was run through a 5Hz low pass filter. The EMG results show a large amounts of co-activation in the TA (Figure S3), which agrees with results from Beck et al. [2020]. Beck et al. [2020] relates the energetic cost to active muscle volume, and find that the SOL active muscle volume can explain the changes in cost. Our modelling results of the SOL support that there is a relation between active muscle volume in the soleus and the changes in the energetic cost, but by measuring the EMG we find there was also activation in the MG and LG (Figure S3), which could be contributing the energetic cost. Additionally, there does appear to be some activation in the proximal leg muscles - VM, VL, BF, and ST (Figure S3). The co-activation here is likely to stabilize the knee, and will be an additional contributor to the energetic cost. It is thus possible that the difference in magnitude of energetic cost between our model and experimental results from Beck et al. [2020] is due to the co-activation in other leg muscles.

Journal of Experimental Biology • Supplementary information

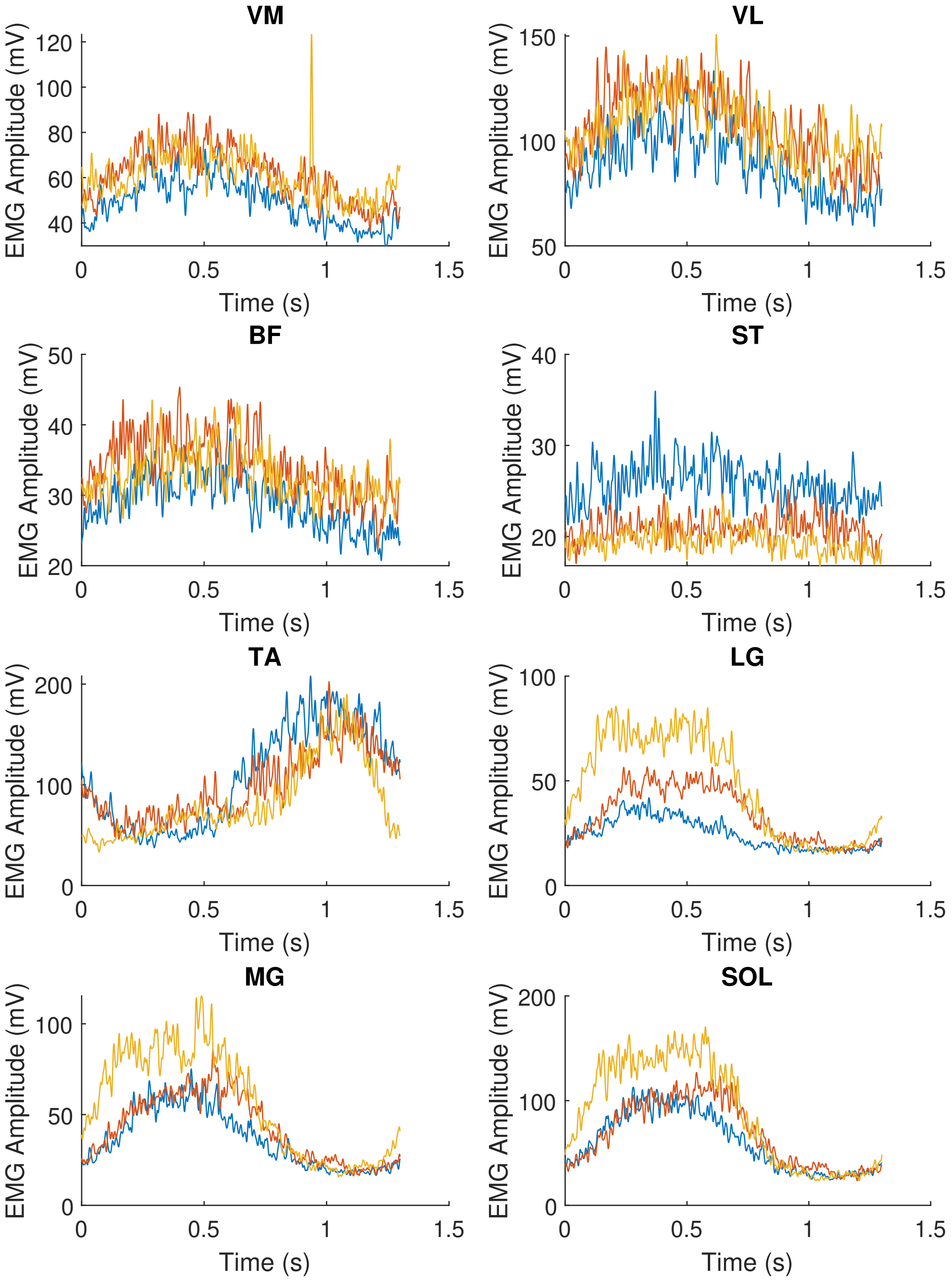

Fig. S3. EMG activity in the leg muscles during plantar flexion averaged over 40 contractions. EMG activity is shown for the 0.6, 0.5, and 0.3 duty cycle conditions, which correspond to the blue, orange, and yellow lines, respectively.

Journal of Experimental Biology • Supplementary information

References

C J Barclay. Mechanical efficiency and fatigue of fast and slow muscles of the mouse. *The Journal of Physiology*, 497(3):781–794, 1996.

C. J. Barclay, R. C. Woledge, and N. A. Curtin. Is the efficiency of mammalian (mouse) skeletal muscle temperature dependent? *The Journal of Physiology*, 588:3819–3831, 10 2010. ISSN 1469-7793. doi: 10.1113/JPHYSIOL.2010.192799.

Owen N. Beck, Jonathan Gosyne, Jason R. Franz, and Gregory S. Sawicki. Cyclically producing the same average muscle-tendon force with a smaller duty increases metabolic rate. *Proceedings of the Royal Society B: Biological Sciences*, 287, 8 2020. ISSN 14712954. doi: 10.1098/RSPB.2020.0431.

Owen N. Beck, Lindsey H. Trejo, Jordyn N. Schroeder, Jason R. Franz, and Gregory S. Sawicki. Shorter muscle fascicle operating lengths increase the metabolic cost of cyclic force production. *Journal of Applied Physiology*, 133:524–533, 9 2022. ISSN 15221601. doi: 10.1152/JAPPLPHYSIOL.00720.2021.

Taylor J.M. Dick, Andrew A. Biewener, and James M. Wakeling. Comparison of human gastrocnemius forces predicted by hill-type muscle models and estimated from ultrasound images. *Journal of Experimental Biology*, 220:1643–1653, 2017. ISSN 00220949. doi: 10.1242/jeb.154807.

G. A. Lichtwark and C. J. Barclay. The influence of tendon compliance on muscle power output and efficiency during cyclic contractions. *Journal of Experimental Biology*, 213:707–714, 3 2010. ISSN 0022-0949. doi: 10.1242/JEB.038026.

J. A. Rall and R. C. Woledge. Influence of temperature on mechanics and energetics of muscle contraction. *American Journal of Physiology - Regulatory Integrative and Comparative Physiology*, 259, 1990. ISSN 00029513. doi: 10.1152/AJPREGU.1990.259.2.R197.

K. W. Ranatunga. Temperature dependence of mechanical power output in mammalian (rat) skeletal muscle. *Experimental Physiology*, 83:371–376, 1998. ISSN 1469-445X. doi: 10.1113/EXPPHYSIOL.1998.SP004120.

Robert Rockenfeller, Michael Günther, Syn Schmitt, and Thomas Götz. Comparative sensitivity analysis of muscle activation dynamics. *Computational and Mathematical Methods in Medicine*, 2015. doi: 10.1155/2015/585409.

Tim J. van der Zee and Arthur D. Kuo. The high energetic cost of rapid force development in muscle. *Journal of Experimental Biology*, 224, 4 2021. ISSN 14779145. doi: 10.1242/JEB.233965/237823.

Journal of Experimental Biology • Supplementary information
